# A radiomics approach based on support vector machine using MR images for preoperative lymph node status evaluation in intrahepatic cholangiocarcinoma

**DOI:** 10.7150/thno.34149

**Published:** 2019-07-09

**Authors:** Lei Xu, Pengfei Yang, Wenjie Liang, Weihai Liu, Weigen Wang, Chen Luo, Jing Wang, Zhiyi Peng, Lei Xing, Mi Huang, Shusen Zheng, Tianye Niu

**Affiliations:** 1 Sir Run Run Shaw Hospital, Zhejiang University School of Medicine, Hangzhou, Zhejiang, China; 2 Institute of Translational Medicine, Zhejiang University, Hangzhou, Zhejiang, China; 3 College of Biomedical Engineering &Instrument Science, Zhejiang University, Hangzhou, Zhejiang, China; 4 Department of Radiology, the First Affiliated Hospital, Zhejiang University School of Medicine, Hangzhou, Zhejiang, China; 5 Department of Radiology, the First Hospital of Ninghai County Medical Centre, Ningbo, Zhejiang, China; 6 Department of Radiation Oncology, Stanford University School of Medicine, Stanford, California, USA; 7 Department of Radiation Oncology, Duke University Medical Center, Durham, USA; 8 Department of Hepatobiliary and Pancreatic Surgery, the First Affiliated Hospital, Zhejiang University School of Medicine, Hangzhou, Zhejiang, China; 9 Engineering Research Center of Cognitive Healthcare of Zhejiang Province

**Keywords:** Radiomics, intrahepatic cholangiocarcinoma, lymph node metastasis

## Abstract

**Purpose**: Accurate lymph node (LN) status evaluation for intrahepatic cholangiocarcinoma (ICC) patients is essential for surgical planning. This study aimed to develop and validate a prediction model for preoperative LN status evaluation in ICC patients.

**Methods and Materials**: A group of 106 ICC patients, who were diagnosed between April 2011 and February 2016, was used for prediction model training. Image features were extracted from T1-weighted contrast-enhanced MR images. A support vector machine (SVM) model was built by using the most LN status-related features, which were selected using the maximum relevance minimum redundancy (mRMR) algorithm. The mRMR method ranked each feature according to its relevance to the LN status and redundancy with other features. An SVM score was calculated for each patient to reflect the LN metastasis (LNM) probability from the SVM model. Finally, a combination nomogram was constructed by incorporating the SVM score and clinical features. An independent group of 42 patients who were diagnosed from March 2016 to November 2017 was used to validate the prediction models. The model performances were evaluated on discrimination, calibration, and clinical utility.

**Results**: The SVM model was constructed based on five selected image features. Significant differences were found between patients with LNM and non-LNM in SVM scores in both groups (the training group: 0.5466 (interquartile range (IQR), 0.4059-0.6985) vs. 0.3226 (IQR, 0.0527-0.4659), *P*<0.0001; the validation group: 0.5831 (IQR, 0.3641-0.8162) vs. 0.3101 (IQR, 0.1029-0.4661), *P*=0.0015). The combination nomogram based on the SVM score, the CA 19-9 level, and the MR-reported LNM factor showed better discrimination in separating patients with LNM and non-LNM, comparing to the SVM model alone (AUC: the training group: 0.842 vs. 0.788; the validation group: 0.870 vs. 0.787). Favorable clinical utility was observed using the decision curve analysis for the nomogram.

**Conclusion**: The nomogram, incorporating the SVM score, CA 19-9 level and the MR-reported LNM factor, provided an individualized LN status evaluation and helped clinicians guide the surgical decisions.

## Introduction

For liver, intrahepatic cholangiocarcinoma (ICC) is the second common malignancy with steadily growing incidence rate, with 5%-56% 5-year survival rate worldwide 1, 2. When diagnosed, about 35% of ICC patients suffer from synchronous lymph node (LN) metastases 1. Lymph node metastasis (LNM) generally indicates a negative prognosis for patients with ICC 3, 4. Accurate preoperative evaluation of LN status could provide crucial information for treatment strategy decisions, especially for lymph node dissection (LND). In current clinical practice, the preoperative LN status in ICC is evaluated mainly based on the morphological features of the lymph nodes by reviewing the medical images preoperatively (for example, size and morphology of lymph nodes, signal changes within lymph nodes, etc.)^5^, ^6^. The prediction accuracy of current LN status evaluation method is often unstable and unsatisfactory.

A common strategy to predict the LN status was developed based on histopathologic findings, such as tumor differentiation and lymphatic invasion. However, the predictors based on this strategy were only available postoperatively. A clinical model built upon the clinical factors including tumor size, pathological differentiation, and tumor boundary could achieve high sensitivity of 96.1%, but the achievable specificity and accuracy were quite low, with a value of 23.0% in specificity and 40.3% in accuracy 7. The above clinical model was reported to be useful in predicting LN status in patients with ICC. Nevertheless, this method was also challenging to apply in clinical practice, because subjectivity may exist in determination of tumor size and tumor boundary, based on clinician's experience and judgment. When the tumor volume was small, or when the tumor boundary was unclear, the situation became exacerbated, and the prediction accuracy could be questionable.

On the other hand, several image-based methods have been proposed 5, 6, 8-10. Seo *et al.* used the standardized uptake value as the LNM image marker based on positron emission tomography (PET) images 9. However, the high cost of PET scan limited the utility in clinical practice. Nanashima *et al.* developed an LN status prediction model by combining CT findings and serum carbohydrate antigen 19-9 level 5. The CT findings were used as image markers, which defined by radiologists according to the node status of hepatoduodenal ligament, common hepatic artery, and para-aorta based on CT images. The model showed a higher prediction accuracy than previous models based on clinical features only, or medical images alone. However, the underlying geometry and texture features of medical images were not fully excavated in these models. A comprehensive model incorporating clinical features and image features is needed.

Radiomics refers to mining the underlying relationships between quantitative image features and pathophysiology characteristics and then developing predictive models for clinical outcomes, such as survival, distant metastases, and molecular characteristics classification 11-15. The use of nomograms has been widely accepted as a reliable tool by incorporating quantitative risk factors for clinical events. Recently, several researchers have developed nomograms for preoperative LN status evaluation in colorectal cancer and bladder cancer by incorporating clinical features and image features 16-18. These nomograms achieved desirable predictive accuracies. The related studies demonstrated the feasibility of using the radiomics method to evaluate the LN status for patients with ICC.

In this study, a support vector machine (SVM) model was developed by using the radiomics method for preoperative LN status evaluation in ICC patients. A nomogram was then constructed by combining the clinical features and LNM probability, which was calculated based on the SVM model with the radiomics features from the MR images. We then investigated the difference in prediction accuracies between the combination model and the SVM model.

## Methods

### Workflow

**Figure 1:**
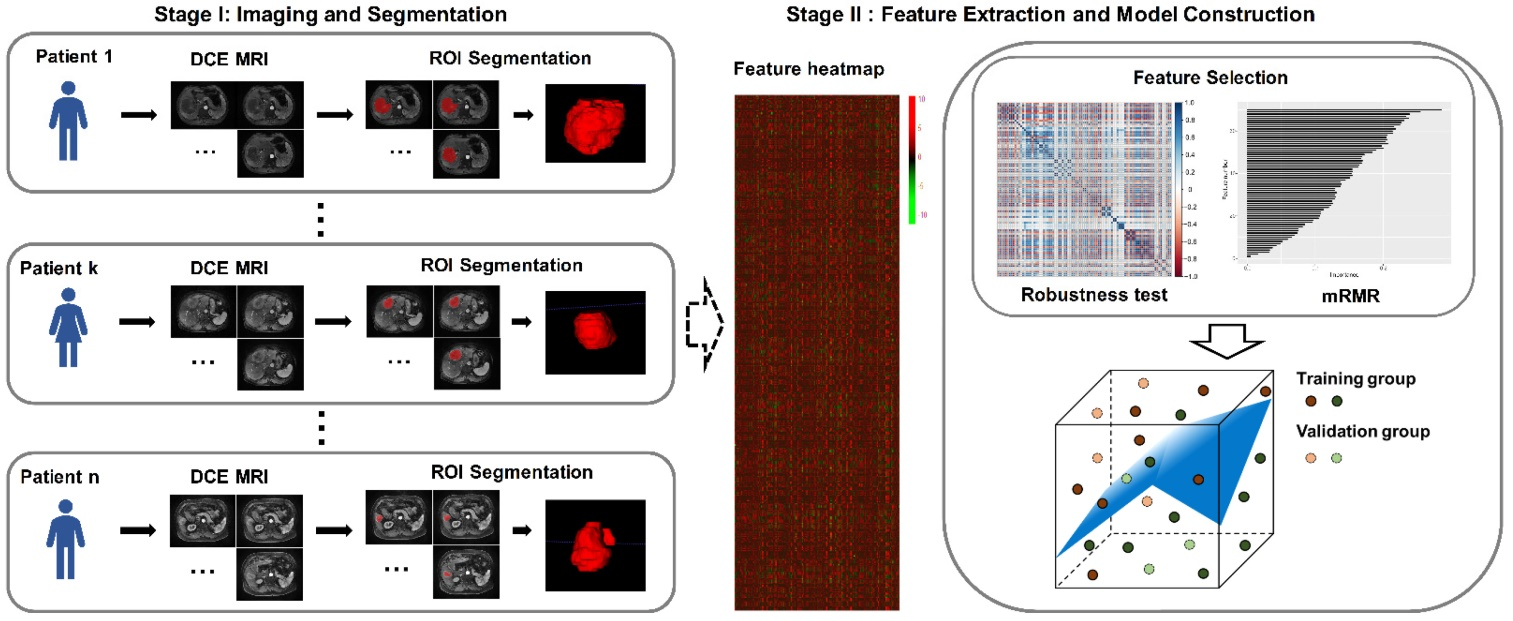
Workflow of this study. The letters k and n are used to number the patients.

**Figure 1** presents the workflow of this study. It includes two major parts: (ⅰ) imaging and segmentation; (ⅱ) feature extraction and model construction. The specific descriptions for these two parts are provided in the next sections.

### Patient Population

The institutional review board of the institution (First Affiliated Hospital, Zhejiang University School of Medicine) approved this study, and the requirement of written informed consent was waived. In this retrospective study, we reviewed clinical records and T1-weighted contrast-enhanced MR images of ICC patients undergoing partial hepatectomy and LND between April 2011 and November 2017. The clinical LN status was obtained based on the clinicopathologic analysis of each patient (X-M Z, a pathologist with more than 10 years of experience in cancer diagnosis). Supplementary Material Ⅰ presents the patient inclusion-exclusion criteria and recruitment pathway. We divided the overall patient population into two independent sub-groups according to the diagnosis time. The first sub-group was used as the training group to test the robustness for image features and conduct the model constructing purpose. This training group consisted of 106 patients diagnosed between April 2011 and February 2016 (53 females and 53 females; 35 to 86 years of age). The second sub-group was used as the validation group to test the proposed model. The validation group involved 42 patients (13 females and 29 females; 40 to 80 years of age) diagnosed between March 2016 and November 2017. All patients underwent pre-treatment T1-weighted contrast-enhanced MRI scans in our institution, since the contrast agent (Gadopentetate dimeglumine) was paramagnetic, and the signal intensity within the tumor would be enhanced after the injection in the images. The tumor detection and characterization of tumor phenotype appeared to be improved compared with the un-enhanced MRI and contrast-enhanced CT. The MRI acquisition parameters are presented in Supplementary Material II.

Baseline clinical features were derived from medical records, including gender, age, cholelithiasis (with or without), hepatitis B (with or without), cirrhosis (with or without), primary hepatic lobe site (left or right) and number of the primary tumors (single or multiple). The serum carbohydrate antigen 19-9 (CA19-9) level (abnormal or normal) and serum carcinoembryonic antigen (CEA) level (abnormal or normal) were achieved with the threshold value of the former 37 u/ml, and the latter 5 ng/ml in our institution. All MR images were evaluated by two experienced abdomen radiologists, both of whom were blind to the actual clinicopathologic results. The definitions for hepatitis B, number of the primary tumors and the MR-reported LNM factor are provided in Supplementary Material Ⅲ. The number of the primary tumors was used as a clinical predictive factor, which referred to the number of solid primary tumors for each patient. To justify the use of baseline clinical features of patients in the training and validation groups, we performed the demographic comparison for each clinical feature between the training group and validation group for patients with LNM and non-LNM, respectively.

### VOI Segmentation and Feature Extraction

We used the ITK-SNAP software to perform a 3D volume of interest (VOI) manual segmentation 19. When multiple tumors were present, the tumor with the largest diameter was used to analyze. The VOI was segmented by two experienced radiologists independently. Radiologist-1 had experience of 12 years in MR images interpretation, and Radiologist-2 had experience of 14 years in MR images interpretation. Radiologist-1 finished the segmentation of patients in the training group only (106 patients). Radiologist-2 finished the segmentation of the overall patient population including the training and validation groups (148 patients). We then obtained two feature sets for the training group (feature set-1 was extracted based on the VOI segmentation of Radiologist-1; feature set-2 from the VOI segmentation of Radiologist-2) and a feature set for the validation group (feature set-3 from the VOI segmentation of Radiologist-2). The feature set-1 was used to perform the model training task. The feature set-2 was used to test the robustness and reproducibility of radiomics features from feature set-1. The feature set-3 was used to evaluate the predictive power of the proposed model.

Image preprocessing was applied before the feature extraction, including image resampling of the arterial phase contrast-enhanced MR images to a 1×1×1 mm^3^ voxel size, and image grey level normalization to a scale of 1 to 32. A total number of 491 image features was extracted for each patient based on the VOI. The feature set included histogram features (number=6), geometry features (number=8), gray level co-occurrence matrix features (number=22), grey-level run-length matrix features (number=13), grey-level size zone matrix features (number=13), neighborhood gray-tone difference matrix features (number=5), and wavelet-based texture features (number=424). These features could characterize intratumor heterogeneity, as well as the underlying tumor genotypes and protein structures 20-26. Supplementary Material Ⅳ provides the specific descriptions for all the radiomics features. The feature extraction procedure was implemented in MATLAB V2017b (MathWorks, Natick, MA, USA).

### Feature Selection and SVM Model Construction

To eliminate the differences in the value scales of the radiomics features, feature normalization was performed before feature selection. For features in the training group, each feature for a specific patient was subtracted by the mean value and divided by standard deviation value from this group. The same normalization method was applied to features in the validation group using the mean values and standard deviation values calculated based on the training group.

Due to the relatively low-dimensional patient sample size and high-dimensional feature size, we then performed feature selection process to select the most LN status-related features to construct an SVM model. Feature selection was performed including two steps. First, we tested the robustness and reproducibility of image features. Since the features were extracted based on the VOIs segmented by radiologists manually, we only used the features that were most robust against the manual segmentation among different radiologists 26. The correlation coefficient for each feature was calculated between the feature set-1 (from Radiologist-1) and feature set-2 (from Radiologist-2) by using the Spearman rank correlation test. Features with correlation coefficients greater than 0.8 were regarded as robust features, since a correlation coefficient of 0.8 indicated a high correlation according to a rule of thumb 26, 27. Second, we applied the maximum relevance minimum redundancy (mRMR) algorithm to assess the relevance and redundancy for each feature 28, 29. The maximum-relevance selection was aimed to select features that had the maximal correlation to the actual LN status. The minimum-redundancy selection ensured that the selected features had the minimal redundancy among each other. By using the mRMR method, the features were ranked according to their relevance-redundancy indexes. The several top features with high-relevance and low-redundancy were used to construct the SVM model by a linear kernel. In addition, we tried several typical feature selection methods, including mRMR, least absolute shrinkage and selection operator (LASSO), Random Forest, Elastic Net, Wilcoxon, and Gini index 30-32. A comparison of these methods was also performed.

To demonstrate the association between the selected features and the actual LN status, we performed univariate analysis and correlation test for each selected features in the training group. An SVM score was calculated by using the SVM model for each patient to reflect the LNM probability. The discrimination measured the capacity of prediction models in separating patients with LNM and non-LNM. The discriminative capability was measured using receiver operating characteristic (ROC) curve, area under the curve (AUC) and prediction accuracy. AUC had a range from 0.5 to 1.0 (0.5 means no discriminative ability and 1.0 means ideal discriminative ability). The AUC was reported with a 95% confidence interval (CI). The prediction accuracy was calculated based on a threshold from a Youden Index, which could classify the patients into the predicted LNM group and non-LNM group according to the SVM score 33, 34. To estimate the prediction error and confidence interval for both groups, we further tested the proposed model using a 10000-iteration bootstrap analysis in both training and validation group 35. For each repetition, we randomly selected a subset of 75% patients from the training group or the validation group (the training group: 80 patients; the validation group: 32 patients) and calculated the corresponding AUC.

### Development and Validation of Combination Nomogram

Multivariable analysis was applied to combine the clinical features and the SVM score with multivariable logistic regression model 17, 36. The clinical features involved gender, age, cholelithiasis status, hepatitis status, cirrhosis status, primary site, number of the primary tumors, CEA level, CA19-9 level, and the MR-reported LNM. Then, a nomogram (called combination nomogram) was generated based on the proposed multivariate model. An LNM probability defined as nomogram score was then calculated for each patient by using the developed combination nomogram. To detect the multi-collinearity among variables in the combination nomogram, the collinearity diagnosis was conducted by calculating the variance inflation factor (VIF) for variables in the combination nomogram 36, 37. The VIF was defined as a ratio of the variance of the model with more than two variables, divided by the variance of the model with the single variable. Variables with VIFs > 10 indicated severity multicollinearities 37. The threshold for the nomogram score was determined and used to classify patients in the validation group. We tested the nomogram by using the overall group including the feature set-2 and feature set-3. In addition, the combination nomogram was also tested using the bootstrap method in both training and validation groups.

The model performances were evaluated in three aspects: discrimination, calibration and clinical utility 17, 24. The discrimination performance was accessed by using ROC, AUC, and prediction accuracy. The calibration was detected by using the calibration curves accompanied by the Hosmer-Lemeshow test (H-L test). The calibration curves measured the consistency between the predicted LNM probability and the actual LNM probability. The H-L test accessed the goodness-of-fit of the prediction models 17, 38. A significant statistic from the H-L test indicated the significant difference between the predicted LNM probability and the actual LNM probability, meaning that the model was poor fitting. For the test of the overall group, we used the Delong test to measure the differences in ROC curves between combination nomogram and the SVM model 39, 40.

The decision curve analysis was applied to measure the clinical utility of models 41, 42. The horizontal axis of the decision curve indicates the threshold probability in the range of 0.0 to 1.0. The vertical axis shows the clinical net benefit values resulted from the prediction models against the threshold probability. The decision curves corresponding to the “treat-all plan” and the “treat-none plan” are plotted as references. The detailed descriptions of the clinical net benefit, the “treat-all plan” and the “treat-none plan” are provided in Supplementary Material Ⅴ. A larger area under the decision curve suggested a better clinical utility.

### Statistical Analysis Procedure

All statistical tests used in this study were executed on MedCalc Statistical Software V15.2.2 (MedCalc Software bvba, Ostend, Belgium) or R software V3.4.1 (R Core Team, Vienna, Austria). Univariate analysis for clinical features was implemented by using the Chi-square test or Mann-Whitney U test, as appropriate. The categorical variable was analyzed using the Chi-square test, such as gender, primary site, number of the primary tumors, CEA level, etc. The continuous variable was analyzed using the Mann-Whitney U test, including age and tumor size. The *P*<0.05 in two-tailed analyses was defined as the statistical significance.

## Results

### Clinical Features

**Table 1** listed the clinical features of patients in the training group and validation group. No statistically significant difference existed in LNM rate (*P* = 0.9210) between the two groups. The LNM rate was defined as the ratio between the number of patients with LNM and the number of patients involved in the certain group. The LNM rate was 44.34% in the training group, and 45.24% in the validation group. While a temporal interval existed between the training and validation groups, there were no significant differences in the baseline clinical features between the training group and the validation group neither for patients with LNM nor non-LNM, justifying their use as the training and validation groups. The detailed results of univariable association analysis were presented in Supplementary Material Ⅵ.

### Feature Selection and SVM Model Construction

Among the 491 image features, 91 features were retained through the robustness and reproducibility test with correlation coefficients greater than 0.8 between the feature set-1 and feature set-2 by using the Spearman rank correlation test. The mRMR based feature selection was used to decrease the redundancy of the feature set and build the optimal subset of complementary predictive features. The five highest mRMR-ranked features were selected to build the SVM model. The calculation formula for the SVM model was provided in Supplementary Material Ⅶ. The selected features were *HLH_GLCM_maxpr*, *LLH_GLCM_sosvh*, *HLL_GLCM_corrm*, *LLL_GLCM_denth* and *HLL_GLSZM_LGZE*. Among the five features, three features of *HLH_GLCM_maxpr*, *LLL_GLCM_denth*, and *HLL_GLSZM_LGZE* showed significant correlation with the actual LN status and significant difference between the patients with LNM and non-LNM in the training group with *P* < 0.05. The univariate analysis and correlation analysis for the selected features were summarized in Table 2. By comparing the prediction performances of different feature selection methods, it was noticed that the mRMR method showed the optimal performance. The specific prediction performances of different methods are summarized in Supplementary Material Ⅷ.

### Validation and Evaluation of SVM Model

**Figure 2:**
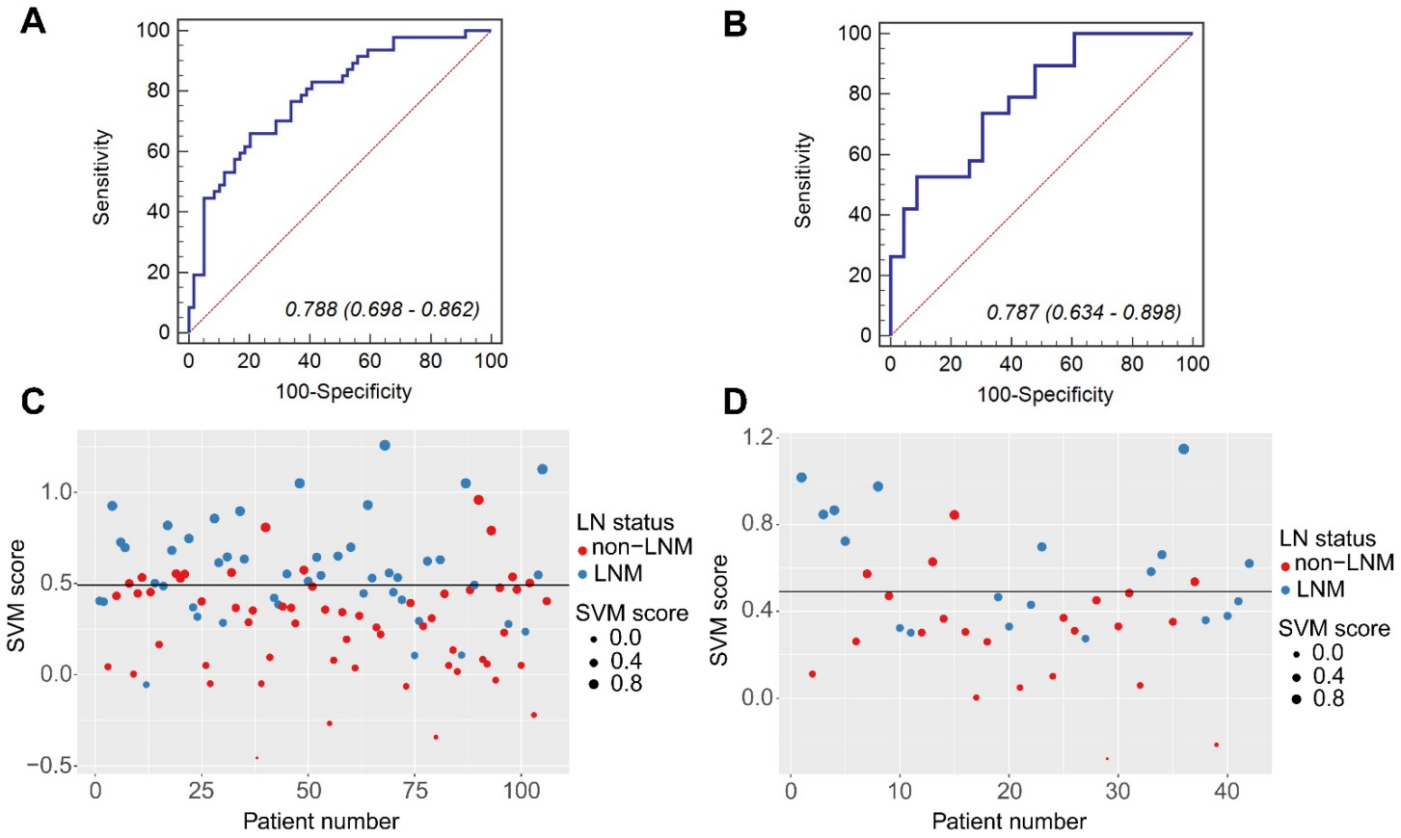
The ROC curves of the SVM model in the training group (A) and the validation group (B). The scatter plots of the SVM scores in the training group (C) and the validation group (D). The blue markers indicate patients with synchronous LNM; the red markers indicate patients with non-LNM. The black horizontal line presents the threshold. Patients with SVM scores higher than 0. 4915 are classified as LNM; patients with scores lower than 0. 4915 are classified as non-LNM.

Significant differences were observed in SVM scores between the patients with synchronous LNM and non-LNM in both groups (the training group: 0.5466 (interquartile range (IQR), 0.4059-0.6985) vs. 0.3226 (IQR, 0.0527-0.4659), *P*<0.0001; the validation group: 0.5831 (IQR, 0.3641-0.8162) vs. 0.3101 (IQR, 0.1029-0.4661), *P* =0.0015). The AUC value was 0.788 (95% CI, 0.698-0.862) for the training group, and 0.787 (95% CI, 0.634-0.898) for the validation group. These values were consistent with the AUC values calculated by using the 10000 times bootstrap analysis in both training and validation groups (mean ± standard deviation; the training group: 0.788±0.027; the validation group: 0.787±0.041). Histograms describing the distributions of AUCs from the bootstrap method for the SVM model were provided in Supplementary Material Ⅸ. By using the Youden Index in the training group, the threshold for the SVM score was defined as 0.4915. By using this threshold, patients with SVM scores higher than 0.4915 were classified as synchronous LNM, while patients with scores lower than 0.4915 were classified as non-LNM. The prediction accuracy was 73.58% for the training group and 69.05% for the validation group. The ROC curves and scatter plots for the SVM score were presented in Figure 2.

### Development of Combination Nomogram

**Figure 3:**
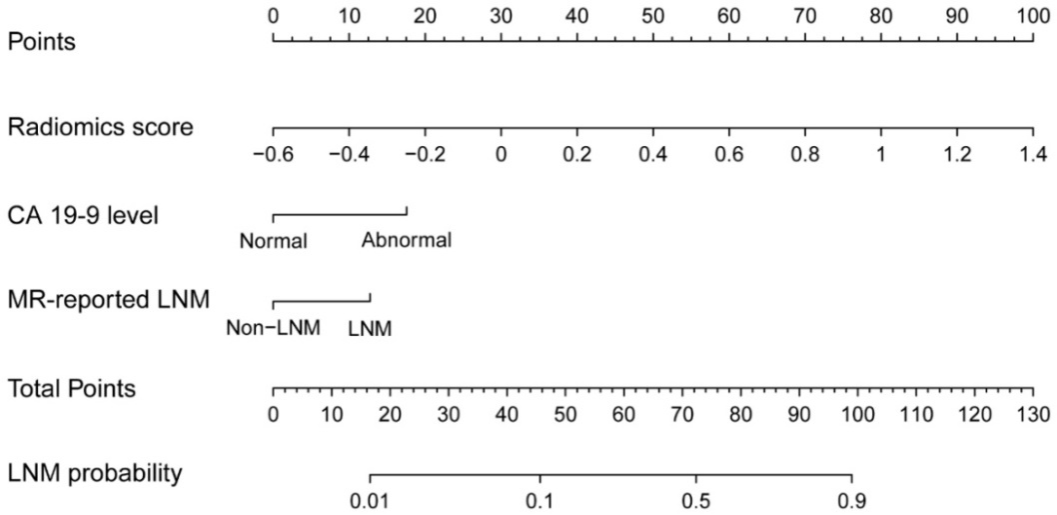
The combination nomogram, combining SVM score, CA 19-9 level, and the MR-reported LNM factor.

In the multivariable analysis, we used the Akaike information criterion (AIC) and the independence analysis to select the optimal feature combination. A combination of the SVM score, CA 19-9 level, and the MR-reported LNM factor was finally selected. The detailed descriptions of the model construction procedure were provided in Supplementary Material Ⅹ. By using the collinearity diagnosis, the VIFs for the SVM score, CA19-9 level, and the MR-reported LNM factor were less than 10 (SVM score: 4.9109; CA19-9 level: 3.7210; MR-reported LNM: 1.9614), indicating no severe collinearity existing in these factors. Using the multivariable analysis, the three factors including the SVM score (*P*<0.0001), CA19-9 level (*P*=0.0081), and the MR-reported LNM factor (*P*=0.0307) were all statistically significant and independent in the training group (Supplementary Material Ⅹ). The combination nomogram was displayed in Figure 3. The calculation formula for the combination nomogram was provided in Supplementary Material Ⅶ.

### Validation and Evaluation of Combination Nomogram

**Figure 4:**
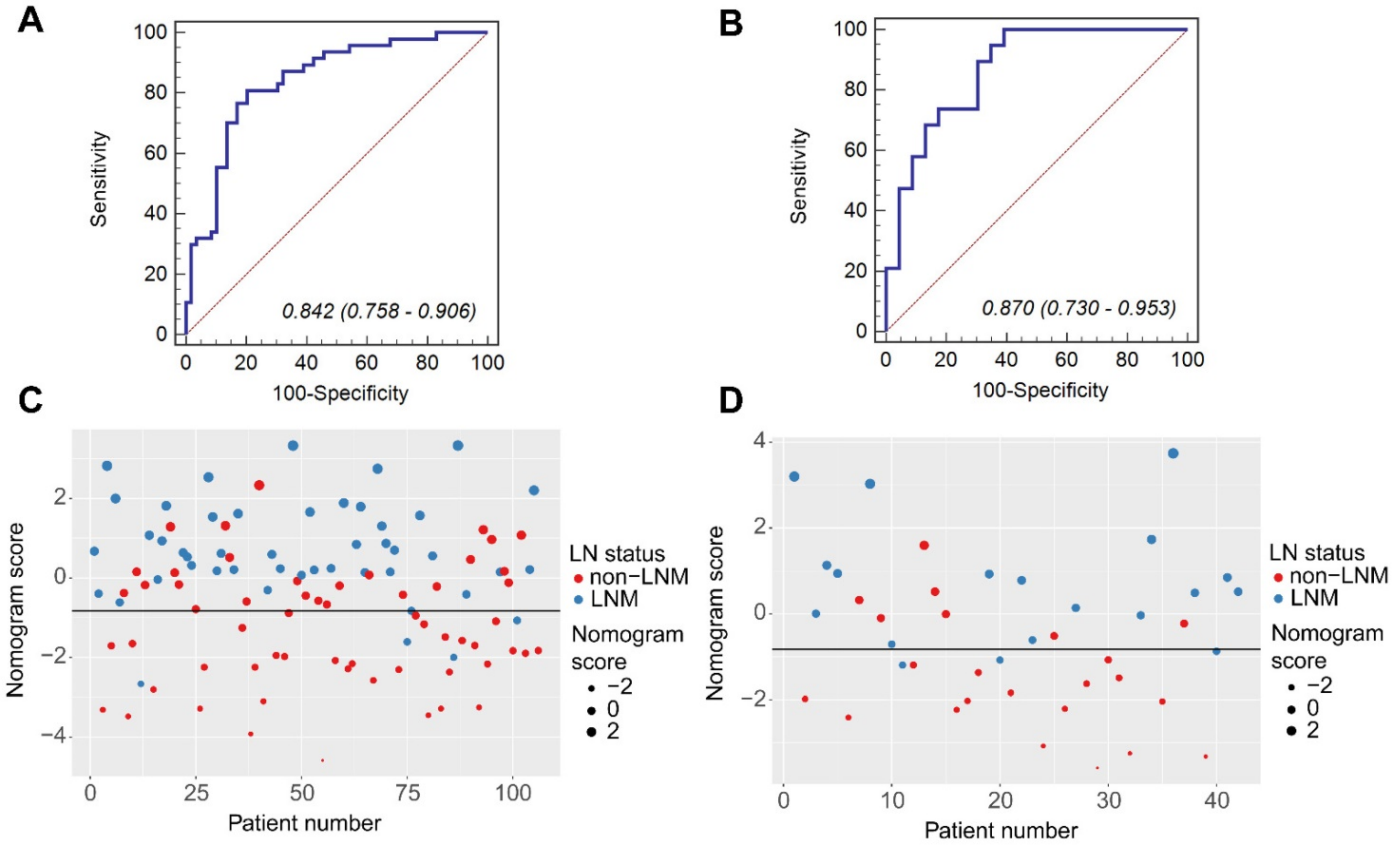
The ROC curves of the combination nomogram in the training group (A) and the validation group (B). The scatter plots for the nomogram score in the training group (C) and the validation group (D). The blue markers indicate patients with synchronous LNM; the red markers indicate patients with non-LNM. The black horizontal line presents the threshold. Patients with nomogram scores higher than -0.8270 are classified as LNM; patients with scores lower than -0.8270 are classified as non-LNM.

**Figure 5:**
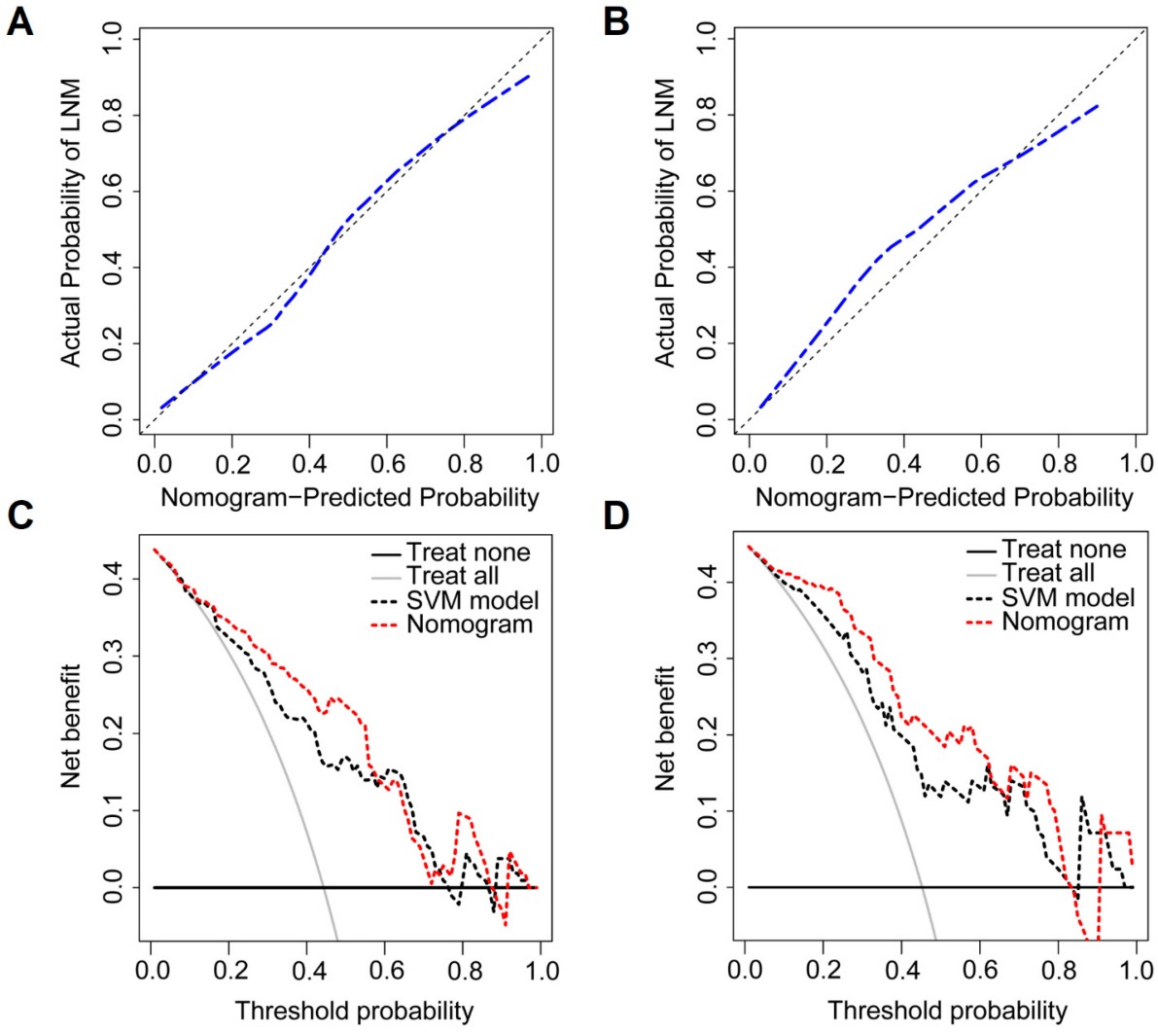
The calibration curves of the combination nomogram in the training group (A) and the validation group (B). Vertical axis: the actual probability of LNM probability; horizontal axis: the nomogram predicted LNM probability; the diagonal line: the perfect prediction with predicted LNM probabilities equal to the actual LNM probabilities. The decision curves of the SVM model and the combination nomogram in the training group (C) and the validation group (D). Vertical axis: the net benefit; horizontal axis: the threshold probability at a range of 0.0 to 1.0. The red and black dotted lines represent the decision curve of the combination nomogram and the SVM model, respectively. The gray line represents the decision curve of the assumption that all patients suffer from LNM; the black line represents the decision curve of the assumption that no patients suffer from LNM.

Compared to patients with synchronous non-LNM, patients with synchronous LNM had higher nomogram scores (the training group: 0.5928 (IQR, 0.1422-1.6073) vs. -1.2560 (IQR, -2.2466- -0.1691), *P*<0.0001; the validation group: 0.5151 (IQR, -0.4691-1.0837) vs. -1.6298 (IQR, -2.2261- -0.3005), *P*<0.0001). The calibration curves demonstrated good consistency between the predicted LNM probability and the actual LNM probability for the combination nomogram in both training and validation groups. For the training group, a non-significant statistic (*P*=0.4650) of the H-L test suggested no significant deviation from an ideal fitting. The AUC value was 0.842 (95% CI, 0.758-0.906). For the validation group, a non-significant statistic of *P*=0.8578 and an AUC of 0.870 (95% CI, 0.730-0.953) were obtained. By using the bootstrap method, the AUC values were generally consistent with that calculated based on the two groups (mean ± standard deviation; the training group: 0.842±0.026; the validation group: 0.869±0.033). Histograms describing the distributions of AUCs from the bootstrap method for the combination nomogram were provided in Supplementary Material Ⅸ. Figure 4 displayed the ROC curves and scatter plots for the nomogram score. The calibration curves for the combination nomogram were provided in Figure 5A-B.

The prediction accuracy for the nomogram was calculated based on the threshold for the nomogram score. By using the Youden Index in the training group, the optimal threshold of -0.8270 was selected in the ROC analysis. Patients with the nomogram scores greater than -0.8270 were predicted as synchronous LNM, while patients with scores lower than -0.8270 were predicted as non-LNM. The prediction accuracy was 72.64% for the training group and 78.57% for the validation group. The decision curves for the combination nomogram and the SVM model were used to evaluate the clinical utilities. In both training and validation groups, the combination nomogram (red) showed a higher area under decision curves than the SVM model (black) (Figure 5C-D). The specific performances of the combination nomogram, the SVM model and the MR-reported LNM factor in both groups were summarized in Table 3.

### Overall Validation of the SVM Model and Combination Nomogram

**Figure 6:**
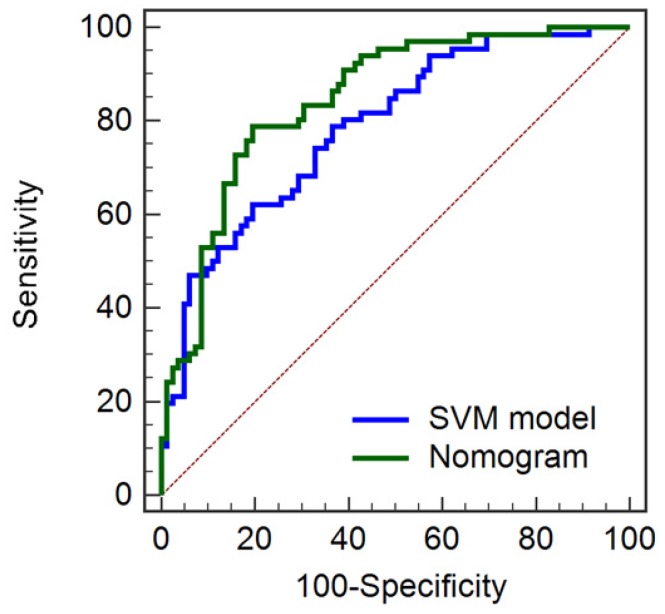
ROC curves for the SVM model and combination nomogram in the overall group.

The prediction models were developed based on training group segmented by Radiologist-1 (feature set-1). To test the robustness and deliverability of the prediction models, we further tested the SVM model, and the combination nomogram using the overall dataset segmented by Radiologist-2 (feature set-2 and feature set-3). The combination nomogram showed better performance (Accuracy, 74.32%; AUC, 0.846 (95% CI, 0.777-0.900); Sensitivity, 87.88%; Specificity, 60.98%) than the SVM model alone (Accuracy, 67.57%; AUC, 0.787 (95% CI, 713-0.850); Sensitivity, 56.06%; Specificity, 78.05%). Further, significant differences from Delong test suggested significant improvements in predictive performances between the combination nomogram and the SVM model (*P*=0.0219). The ROC curves of the combination nomogram and the SVM model for the overall group were shown in Figure 6.

## Discussion

We developed and validated a nomogram by using radiomics approach for LN status preoperative evaluation in this study. The combination nomogram was constructed by incorporating the SVM score from the radiomics method and two clinical features of CA19-9 level and the MR-reported LNM factor. SVM score was an LNM probability calculated from the SVM model, which was developed based on five selective image features. The combination nomogram outperformed the SVM model in both training and validation groups (the training group: 0.842 vs. 0.788; the validation group 0.870 vs. 0.787). Thus, the favorable preoperative LN status prediction power of the proposed non-invasive method made it a potential preoperative evaluation tool in clinical practice.

The manual process of tumor segmentation and the reproducibility of radiomics features are the most debatable aspects in the radiomics analysis. Subjectivity in the determination of tumor volume and tumor boundary would occur. The uncertainties in tumor segmentation adversely affect the reproducibility of radiomics features 43. A recent study investigating the robustness and reproducibility of radiomics features in different MRI sequences suggested that radiomics features extracted from T1-weighted images should be used with care, and only those reproducible features should be selected in building a radiomics model 44. In this study, all patients were scanned in the same MRI scanner with liver acceleration volume acquisition (LAVA) sequence. The tumor segmentations were performed by two radiologists independently. Furthermore, we tested the robustness and reproducibility of image features by using two feature sets extracted based on the segmentations of the two radiologists. The five selected features and the proposed SVM model were found to be robust against tumor segmentation.

Note that all the selected five image features used in the SVM model were wavelet features. These features were extracted from images decomposed by undecimated 3D wavelet transforms. The wavelet transformation was a multiscale image analysis method by splitting the 3D image data into different frequency components along three axes. Fine and coarse texture extracted from the wavelet decomposed images could further present the spatial heterogeneity at multiple scales within tumor regions 45. By using the correlation analysis, three out of the five features showed significant correlation with the actual LN status with *P*<0.05. The possible reason was that the wavelet features had underlying associations with clinicopathology and tumor lymphatic system invasion. This observation was consistent with previous studies which used wavelet-based features in the radiomics models 46-48. Recently, a study developed a prediction model to preoperatively differentiate pathological grades in patients with pancreatic neuroendocrine tumors 46. The prediction model was constructed using eight image features, and seven out of them were wavelet features. A radiomics study employed machine-learning methods to predict histologic subtypes for patients with lung cancer. Four out of five features included in the model were wavelet features 48. These studies confirmed that wavelet features are important imaging biomarkers for predicting the phenotype of tumors because they are closely related to the biological behavior of tumors.

In this study, CA19-9 level was served as an independent marker in prognosis stratification in patients with ICC, which was consistent with the previous studies 49. In 2001, Jiang *et al.* proposed a clinical feature based prognostic score to accurately predict the prognosis for patients with ICC regardless of resection status, in which CA 19-9 was the only laboratory marker. It was also used as an independent predictive factor in prognosis evaluation in ICC patients with partial excision. More importantly, a study reported that the CA 19-9 level was also associated with the tumor progression of ICC 50. Two recent studies both reported that the preoperative abnormal level of CA 19-9 was valuable in preoperative LN status evaluation 51, 52. Similarly, the CA19-9 level was used as an independent predictor in the combination nomogram in this study, which also could improve the predictive power of the SVM model.

The proposed preoperative LN status prediction model has potential in assisting clinicians in making the effective surgical decision for patients with ICC. Although a series of studies had reported that LNM was highly correlative to the prognosis of ICC, the benefit of lymph node dissection (LND) is still controversial 53, 54. de Jong *et al.* found that among patients who underwent routine LND, patients with LNM showed a worse median survival 55. Meng *et al.* revealed that LND only benefited a subset of patients with a moderate survival benefit of about five months 56. LNM-related prognostic stratification is a significant clinical problem in the management of ICC patients. Accurate preoperative LN status evaluation represents a key step in individualized and precision treatment of ICC patients.

Our study still had several limitations. Firstly, the patient population was collected from a single institution retrospectively. A total of 106 patients was enrolled in the training group, and 42 patients in the validation group. To evaluate the sample size for the validation group, we performed a power analysis based on the LNM rates of the training dataset and the validation dataset. Normally, a power value greater than 0.8 suggests a sufficient sample size 57, 58. Our estimated power value was 0.85 for the current study. Thus, the sample sizes for the training and the validation groups were sufficient, meaning that the result and conclusion of this study were statistically significant. In the future, we will test the proposed model with multi-center and larger sample size. Secondly, we did not incorporate genomic characteristics in this study. Recently, increased researches with gene markers had been proposed to detect LNM in patients with ICC, such as VEGF and EGFR 59. Though it might be an interesting study to combine genomics and radiomics analysis, it has not yet been determined how to incorporate genomic characteristics, image features, and clinical features together. Thirdly, because the diffusion-weighted imaging (DWI) sequences were altered several times during the long-time span of the study, we used only T1-weighted arterial phase MR images to mitigate any possible adverse effect caused by the changes in the DWI sequences and enhance the stability and robustness of the predictive model.

## Conclusions

This study developed and validated a combination nomogram for the LN status preoperative evaluation in ICC patients. The combination nomogram developed using SVM score, CA19-9 level, and the MR-reported LNM factor showed better prediction accuracy than the MR-reported LNM factor and the SVM model alone. The proposed model could be used for individualized LN status evaluation and would help clinicians guide the surgical decisions. Multi-institution retrospective and prospective validation studies should be implemented before the practical application in the future clinical surgical plan determination.

## Supplementary Material

Supplementary information, figures and tables.

## Figures and Tables

**Table 1:** Patients and preoperative clinical feature

Clinical features	Training group (n=106)		*P*	Validation group (n=42)		*P*
	LNM	non-LNM		LNM	non-LNM	
Age (Mean± SD)	58.02 ± 10.54	60.05 ± 8.52	0.2755	55.93 ± 15.25	60.34 ± 7.17	0.4662
Range	(35, 77)	(39, 86)		(40, 80)	(43, 76)	
Gender			0.8450			0.5547
Male	23	30		5	8	
Female	24	29		14	15	
Primary hepatic lobe site			0.6683			0.2479
Left	30	40		14	13	
Right	17	19		5	10	
Number of the primary tumors			0.0013			0.1536
Single	30	53		12	19	
Multiple	17	6		7	4	
Hepatitis			0.7065			0.6179
Without	35	42		15	24	
With	12	17		4	9	
Cirrhosis			0.4274			0.6673
Without	46	56		18	21	
With	1	3		1	2	
Cholelithiasis			0.2506			0.7030
Without	38	42		15	17	
With	9	17		4	6	
CA19-9			0.0086			0.0339
Normal	10	27		7	16	
Abnormal	37	32		12	7	
CEA			0.0674			0.0143
Normal	29	46		10	20	
Abnormal	18	13		9	3	
MR-reported LNM			0.0012			0.0251
Negative	17	40		5	14	
Positive	30	19		14	9	

Note: LNM, lymph node metastasis; CA19-9, serum carbohydrate antigen 19-9; CEA, serum carcinoembryonic antigen; SD, standard deviation.

**Table 2:** Univariate analysis and correlation test for radiomics features used in the SVM model for the training group

Radiomics features	Training group (n=106)		*P*	Correlation coefficient	*P*
	LNM	non-LNM			
HLH_GLCM_maxpr	0.2854 (0.2651 to 0.3175)	0.2665(0.2425 to 0.2805)	0.0164	0.2343	0.0156
LLH_GLCM_sosvh	1.0462 (0.9128 to 1.1260)	1.1206 (0.9829 to 1.1929)	0.0963	-0.1623	0.0965
HLL_GLCM_corrm	-0.0178 (-0.0212 to -0.0152)	-0.0146 (-0.0175 to -0.0115)	0.0629	-0.1815	0.0626
LLL_GLCM_denth	2.7902 (2.7389 to 2.8908)	2.9404 (2.8816 to 2.9863)	0.0014	-0.3112	0.0012
HLL_GLSZM_LGZE	0.0013 (0.0010 to 0.0014)	0.0018 (0.0014 to 0.0023)	0.0028	0.2920	0.0024

Note: The univariate analysis for radiomics features was applied by using the Mann-Whitney U test. The correlation between radiomics features and the LN status was applied by using the Spearman rank correlation test. All features were reported as median and 95% confidence interval.

**Table 3:** Performances of the SVM model, combination nomogram and MR-reported LNM

Models	Training group						Validation group				
	Accuracy	AUC (95%CI)	Sensitivity	Specificity	S. E.		Accuracy	AUC (95%CI)	Sensitivity	Specificity	S. E.
SVM score	73.58%	0.788 (0.698 - 0.862)	65.96%	79.66%	0.0441		69.05%	0.787 (0.634 - 0.898)	52.63%	91.30%	0.0695
Combination model	72.64%	0.842 (0.758 - 0.906)	89.36%	57.63%	0.0387		78.57%	0.870 (0.730 - 0.953)	89.47%	69.57%	0.0540
MR-reported LNM	66.04%	0.658 (0.560 - 0.748)	63.83%	67.80%	0.0469		66.67%	0.673 (0.511 - 0.809)	73.68%	60.87%	0.0735

Note: SVM, support vector machine; S.E., standard error; CI, confidence interval.

## Abbreviations

ICC: intrahepatic cholangiocarcinoma
LNM: lymph node metastasis
SVM: support vector machine
mRMR: maximum relevance minimum redundancy
LASSO: least absolute shrinkage and selection operator
ROC: receiver operating characteristic
AUC: area under the curve
CI: confidence interval
IQR: interquartile range
VOI: volume of interest
AIC: Akaike information criterion
VIF: variance inflation factor
LND: lymph node dissection
CEA: serum carcinoembryonic antigen
CA 19-9: serum carbohydrate antigen 19-9
