# Supplementary material for: A radiomics approach based on support vector machine using MR images for preoperative lymph node status evaluation in intrahepatic cholangiocarcinoma
Source: Theranostics. 2019 Jul 9;9(18):5374–85. doi: 10.7150/thno.34149 (PMC6691572; doi:10.7150/thno.34149)
Supplement: Supplementary file 1 — Supplementary information, figures and tables. [file thnov09p5374s1.pdf]

Supplementary Material

A radiomics approach based on support vector machine using MR images for preoperative lymph node status evaluation in intrahepatic cholangiocarcinoma

Authors

Lei Xu*, Pengfei Yang*, Wenjie Liang*, Weihai Liu, Weigen Wang, Chen Luo, Jing Wang, Zhiyi Peng†, Lei Xing, Mi Huang†, Shusen Zheng†, Tianye Niu†

I. The patient inclusion and exclusion criteria

II. MR acquisition parameters

III. Determination of hepatitis B, number of the primary tumors, and the MR-reported LNM factor

IV. The feature set developed in this study

V. The detailed descriptions of clinical net benefit, the “treat-all plan”, and the “treat-none plan”

VI. Demographic comparison of baseline clinical features between the training and validation groups

VII. Calculation formulas for SVM model and combination nomogram

VIII. Predictive performances of different feature selection methods

IX. Histograms regarding the distributions of AUCs for the SVM model and combination nomogram

X. The multivariable analysis for model construction

I. The patient inclusion and exclusion criteria

The patient inclusion criteria included the following: (1) All patients were surgically resected with pathologically confirmed ICC. (2) Lymph node dissection was performed during operation. (3) T1-weighted contrast-enhanced MRI scan was performed within one month before the operation. (4) Preoperative clinical records were complete.

The patient exclusion criteria included the following: (1) The disease was diagnosed to be mixed hepatocellular cholangiocarcinoma. (2) The patient underwent chemotherapy before contrast-enhanced MRI scan.

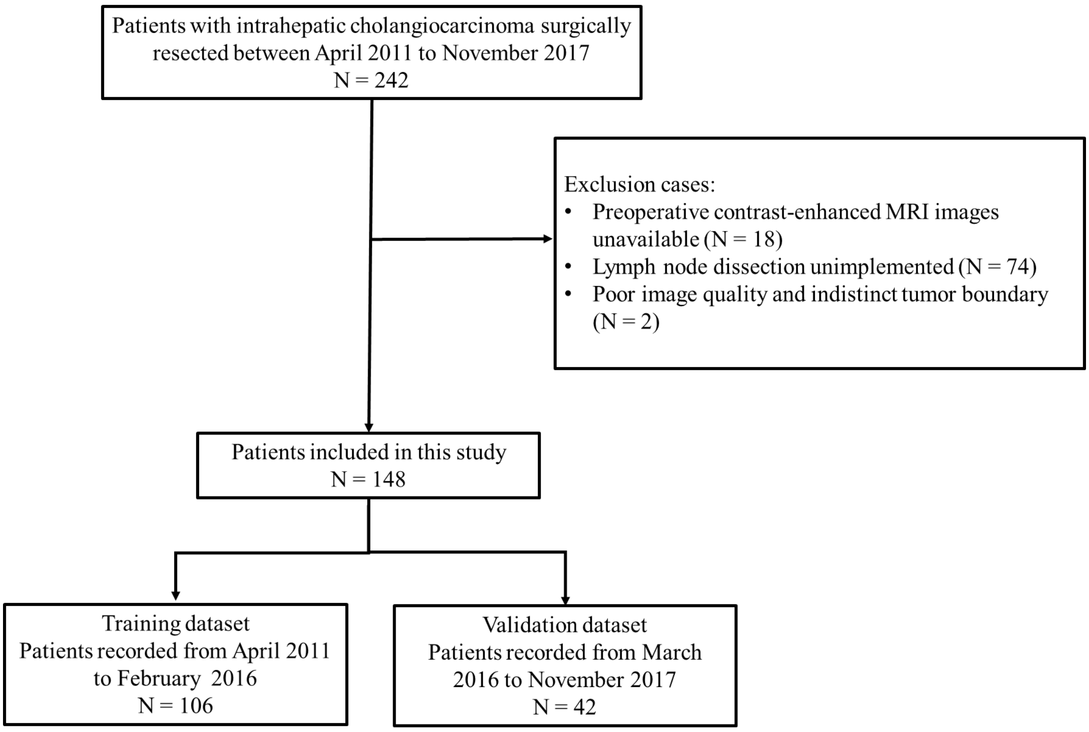

Figure S1. Patient data inclusion/exclusion pathway

II. MR acquisition parameters

All patients underwent preoperative abdominal T1-weighted contrast-enhanced MRI scans. The MRI scans were performed on an MRI scanner (3.0T, GE Medical Systems, Milwaukee, USA) with liver acceleration volume acquisition (LAVA) sequence. By using the abdominal coil, the scan range was defined from the diaphragmatic dome to the lower pole of the kidney. The contrast-enhanced MR imaging acquisition

parameters included: flip angle: 10°; repetition time: 2.75-3.20 ms; echo time: 1.25-1.50 ms; reverse time: 5 ms; bandwidth: 390.63-488.28 kHz; field of view: 380×304 mm; pixel spacing: 0.7031-0.8203 mm; slice thickness: 4-6 mm. Each patient was rapid intravenous injected with 15 ml of Gadopentetate Dimeglumine (2.5 ml/s). The arterial phase was scanned at 14s, then portal vein phase and delayed phase were 55s and 120s after the injection, respectively. Finally, we used the T1-weighted arterial phase enhanced MR images in this radiomics study.

III. Determination of hepatitis B, number of the primary tumors, and the MR-reported LNM factor

All patients involved in this study were diagnosed with chronic hepatitis B. We divided the patients into cirrhosis group (F4) and non-cirrhosis group (F0-3), according to their clinical and liver imaging characteristics. Due to the absence of liver biopsy data, we did not perform accurate liver fibrosis grading.

We used the terminology "number of the primary tumors (single or multiple)" to refer to the case with the number of solid primary tumors. "Single" refers to the case with only 1 solid tumor, while "multiple" refers to the case with the number of solid tumors more than 2.

The MR-reported LNM factor was defined by an agreement of 3 radiologists based on the preoperative MR images. The presence of the maximum short-axis diameter of regional LN ≥ 10mm and/or lymph node margin hyperintense in diffusion MRI images, and/or marginal enhancement was scored as positive LNM, while the absence of enlarged or lymph node margin hyperintense or marginal enhancement was scored as negative LNM, consistent with the definition for LN status evaluation criteria in most previous studies.

IV. The feature set developed in this study

In this study, a number of 491 image features was extracted for each patient. These features comprised of four groups; the detailed descriptions of the image features were provided in Table S1-S4. The histogram

statistics features described the voxel intensities statistical distribution within the tumors. The geometry features described the 3D volume and shape characteristics of the tumors. The texture features described the spatial intensity correlation and distributions of the voxels.

The gray-level co-occurrence matrix (GLCM) is a $N_g \times N_g$matrix defined as $C(m,n;\delta,\alpha)$, where m and n represent gray levels, $\delta(dx,dy)$ is the given distance and α indicates the certain direction which has 13 potential value for 3-dimension. The entry $C(m,n)$ represents the repetition of the correlation of the gray levels m and n . N_g represents the maximum gray level value within the volume of interest (VOI). The gray-level run length matrix (GLRLM) is used to quantify run length matrices within the VOI. It is a $N_g \times N_g$matrix defined as $R(m,n|\theta)$. The element $R(m,n)$ describes the frequency value that the VOI includes a run of length m, consisting of points of gray level n in the certain direction θ. The gray level size zone matrix (GLSZM) is used to quantify size zone matrices within the VOI. It is a $N_g \times N_g$ matrix defined as $S(m,n)$. The element $S(m,n)$ specifies the frequency of block of size n with the gray level m. The neighborhood gray-tone difference matrix (NGTDM) is a column matrix of N_g. It is a sum of the absolute difference values between central voxel and the average value of its neighborhood. The neighborhood is defined as the certain distance of 2 voxels. The detailed feature names and abbreviations are presented below. The feature extraction program was implemented based on MATLAB (Version 2017b; MathWorks, Natick, MA, USA).

Table S1 Histogram feature

**Histogram feature**	Feature names and abbreviations
	Variance,<br>Skewness,<br>Kurtosis,<br>Mean,<br>Energy,<br>Entropy

Table S2 Geometry features

**Geometry feature**	Feature names and abbreviations
	Max Diameter<br>Uniformity,<br>Surface Volume Ratio(SVR),<br>Compactness1(Cpt1),<br>Compactness2(Cpt2),

	Surface Area,<br>Spherical Disproportion(SphDisp),<br>Sphericity

Table S3 Texture features

**Feature type**	Feature names and abbreviations
**GLCM**<br>**(Grey-level co-occurrence matrix)**	Autocorrelation(autoc),<br>Contrast(contr),<br>Correlation(corrm),<br>Correlation2(corrp),<br>Cluster Prominence(cprom),<br>Cluster Shade(cshad),<br>Dissimilarity(dissi),<br>Energy(energ),<br>Entropy(entro),<br>Homogeneity(homom),<br>Homogeneity2(homop),<br>Maximum probability(maxpr),<br>Sum of squares Variance(sosvh),<br>Sum average(savgh),<br>Sum variance(svarh),<br>Sum entropy(senth),<br>Difference variance(dvarh),<br>Difference entropy(denth),<br>Information measure of correlation1(inf1h),<br>Information measure of correlation2(inf2h),<br>Inverse difference normalized (INN) (indnc),<br>Inverse difference moment normalized(idmnc)
**GLRLM**<br>**(Grey-level run-length matrix)**	Short Run Emphasis (SRE),<br>Long Run Emphasis (LRE),<br>Grey-Level Non-uniformity (GLN),<br>Run-Length Non-uniformity (RLN),<br>Run Percentage (RP),<br>Low Grey-Level Run Emphasis (LGRE),<br>High Grey-Level Run Emphasis (HGRE),<br>Short Run Low Grey-Level Emphasis (SRLGE),<br>Short Run High Grey-Level Emphasis (SRHGE),<br>Long Run Low Grey-Level Emphasis (LRLGE),<br>Long Run High Grey-Level Emphasis (LRHGE),<br>Grey-Level Variance (GLV),<br>Run-Length Variance (RLV)
**GLSZM**<br>**(Grey-level size zone matrix)**	Small Zone Emphasis (SZE),<br>Large Zone Emphasis (LZE),<br>Grey-Level Non-uniformity (GLN),<br>Zone-Size Non-uniformity (ZSN),<br>Zone Percentage (ZP),<br>Low Grey-Level Zone Emphasis (LGZE),<br>High Grey-Level Zone Emphasis (HGZE),<br>Small Zone Low Grey-Level Emphasis (SZLGE),<br>Small Zone High Grey-Level Emphasis (SZHGE),<br>Large Zone Low Grey-Level Emphasis (LZLGE),<br>Large Zone High Grey-Level Emphasis (LZHGE),<br>Grey-Level Variance (GLV),<br>Zone-Size Variance (ZSV)
**NGTDM**<br>**(Neighbourhood grey-tone difference matrix)**	Coarseness,<br>Contrast,<br>Busyness,<br>Complexity,<br>Strength

Wavelet features: We use the discrete undecimated wavelet transform for decomposing the original

images. The high-pass and low-pass wavelet functions were used in three axials; then, the original image could be decomposed into eight decompositions. We marked the original 3D images as G, the high-pass wavelet function as H and the low-pass wavelet function as L. Them, the decompositions could be express as G_{LLL}, G_{LLH}, G_{LHL}, G_{HLL}, G_{LHH}, G_{HLH}, G_{HHL}, G_{HHH}. Specificity, the decomposition G_{LHL} indicated that the original image was processed by using a low-pass filter, a high-pass filter and a low-pass filter in the x-axis, y-axis and z-axis, respectively. Based on these 8 decomposed images, histogram and textural features are extracted again. A number of 424 features could be obtained through wavelet transform. The filters used for wavelet transform satisfy the perfect reconstruction conditions.

Table S4 Wavelet features

**Wavelet type**	Feature names
**LLL (low, low, low)**	LLL_GLCM_autoc, LLL_GLCM_contr, LLL_GLCM_corrm, LLL_GLCM_corrp, LLL_GLCM_cprom, LLL_GLCM_cshad, LLL_GLCM_dissi, LLL_GLCM_energ, LLL_GLCM_entro, LLL_GLCM_homom, LLL_GLCM_homop, LLL_GLCM_maxpr, LLL_GLCM_sosvh, LLL_GLCM_savgh, LLL_GLCM_svarh, LLL_GLCM_senth, LLL_GLCM_dvarh, LLL_GLCM_denth, LLL_GLCM_inf1h, LLL_GLCM_inf2h, LLL_GLCM_indnc, LLL_GLCM_idmnc, LLL_GLRLM_SRE, LLL_GLRLM_LRE, LLL_GLRLM_GLN, LLL_GLRLM_RLN, LLL_GLRLM_RP, LLL_GLRLM_LGRE, LLL_GLRLM_HGRE, LLL_GLRLM_SRLGE, LLL_GLRLM_SRHGE, LLL_GLRLM_LRLGE, LLL_GLRLM_LRHGE, LLL_GLRLM_GLV, LLL_GLRLM_RLV, LLL_GLSZM_SZE, LLL_GLSZM_LZE, LLL_GLSZM_GLN, LLL_GLSZM_ZSN, LLL_GLSZM_ZP, LLL_GLSZM_LGZE, LLL_GLSZM_HGZE, LLL_GLSZM_SZLGE, LLL_GLSZM_SZHGE, LLL_GLSZM_LZLGE, LLL_GLSZM_LZHGE, LLL_GLSZM_GLV, LLL_GLSZM_ZSV, LLL_Coarseness, LLL_Contrast, LLL_Busyness, LLL_Complexity, LLL_Strength
**LLH (low, low, high)**	LLH_GLCM_autoc, LLH_GLCM_contr, LLH_GLCM_corrm, LLH_GLCM_corrp, LLH_GLCM_cprom, LLH_GLCM_cshad, LLH_GLCM_dissi, LLH_GLCM_energ), LLH_GLCM_entro, LLH_GLCM_homom, LLH_GLCM_homop, LLH_GLCM_maxpr, LLH_GLCM_sosvh, LLH_GLCM_savgh, LLH_GLCM_svarh, LLH_GLCM_senth, LLH_GLCM_dvarh, LLH_GLCM_denth, LLH_GLCM_inf1h, LLH_GLCM_inf2h, LLH_GLCM_indnc, LLH_GLCM_idmnc, LLH_GLRLM_SRE, LLH_GLRLM_LRE, LLH_GLRLM_GLN, LLH_GLRLM_RLN, LLH_GLRLM_RP, LLH_GLRLM_LGRE, LLH_GLRLM_HGRE, LLH_GLRLM_SRLGE, LLH_GLRLM_SRHGE, LLH_GLRLM_LRLGE, LLH_GLRLM_LRHGE, LLH_GLRLM_GLV, LLH_GLRLM_RLV, LLH_GLSZM_SZE, LLH_GLSZM_LZE, LLH_GLSZM_GLN, LLH_GLSZM_ZSN, LLH_GLSZM_ZP, LLH_GLSZM_LGZE, LLH_GLSZM_HGZE, LLH_GLSZM_SZLGE, LLH_GLSZM_SZHGE, LLH_GLSZM_LZLGE, LLH_GLSZM_LZHGE, LLH_GLSZM_GLV, LLH_GLSZM_ZSV, LLH_Coarseness, LLH_Contrast, LLH_Busyness, LLH_Complexity, LLH_Strength
**LHL (low, high, low)**	LHL_GLCM_autoc, LHL_GLCM_contr, LHL_GLCM_corrm, LHL_GLCM_corrp, LHL_GLCM_cprom, LHL_GLCM_cshad, LHL_GLCM_dissi, LHL_GLCM_energ), LHL_GLCM_entro, LHL_GLCM_homom, LHL_GLCM_homop, LHL_GLCM_maxpr, LHL_GLCM_sosvh, LHL_GLCM_savgh, LHL_GLCM_svarh,

	LHL_GLCM_senth, LHL_GLCM_dvarh, LHL_GLCM_denth, LHL_GLCM_inf1h, LHL_GLCM_inf2h, LHL_GLCM_indnc, LHL_GLCM_idmnc, LHL_GLRLM_SRE, LHL_GLRLM_LRE, LHL_GLRLM_GLN, LHL_GLRLM_RLN, LHL_GLRLM_RP, LHL_GLRLM_LGRE, LHL_GLRLM_HGRE, LHL_GLRLM_SRLGE, LHL_GLRLM_SRHGE, LHL_GLRLM_LRLGE, LHL_GLRLM_LRHGE, LHL_GLRLM_GLV, LHL_GLRLM_RLV, LHL_GLSZM_SZE, LHL_GLSZM_LZE, LHL_GLSZM_GLN, LHL_GLSZM_ZSN, LHL_GLSZM_ZP, LHL_GLSZM_LGZE, LHL_GLSZM_HGZE, LHL_GLSZM_SZLGE, LHL_GLSZM_SZHGE, LHL_GLSZM_LZLGE, LHL_GLSZM_LZHGE, LHL_GLSZM_GLV, LHL_GLSZM_ZSV, LHL_Coarseness, LHL_Contrast, LHL_Busyness, LHL_Complexity, LHL_Strength
**HLL (high, low, low)**	HLL_GLCM_autoc, HLL_GLCM_contr, HLL_GLCM_corrm, HLL_GLCM_corrp, HLL_GLCM_cprom, HLL_GLCM_cshad, HLL_GLCM_dissi, HLL_GLCM_energ), HLL_GLCM_entro, HLL_GLCM_homom, HLL_GLCM_homop, HLL_GLCM_maxpr, HLL_GLCM_sosvh, HLL_GLCM_savgh, HLL_GLCM_svarh, HLL_GLCM_senth, HLL_GLCM_dvarh, HLL_GLCM_denth, HLL_GLCM_inf1h, HLL_GLCM_inf2h, HLL_GLCM_indnc, HLL_GLCM_idmnc, HLL_GLRLM_SRE, HLL_GLRLM_LRE, HLL_GLRLM_GLN, HLL_GLRLM_RLN, HLL_GLRLM_RP, HLL_GLRLM_LGRE, HLL_GLRLM_HGRE, HLL_GLRLM_SRLGE, HLL_GLRLM_SRHGE, HLL_GLRLM_LRLGE, HLL_GLRLM_LRHGE, HLL_GLRLM_GLV, HLL_GLRLM_RLV, HLL_GLSZM_SZE, HLL_GLSZM_LZE, HLL_GLSZM_GLN, HLL_GLSZM_ZSN, HLL_GLSZM_ZP, HLL_GLSZM_LGZE, HLL_GLSZM_HGZE, HLL_GLSZM_SZLGE, HLL_GLSZM_SZHGE, HLL_GLSZM_LZLGE, HLL_GLSZM_LZHGE, HLL_GLSZM_GLV, HLL_GLSZM_ZSV, HLL_Coarseness, HLL_Contrast, HLL_Busyness, HLL_Complexity, HLL_Strength
**HHL (high, high, low)**	HHL_GLCM_autoc, HHL_GLCM_contr, HHL_GLCM_corrm, HHL_GLCM_corrp, HHL_GLCM_cprom, HHL_GLCM_cshad, HHL_GLCM_dissi, HHL_GLCM_energ), HHL_GLCM_entro, HHL_GLCM_homom, HHL_GLCM_homop, HHL_GLCM_maxpr, HHL_GLCM_sosvh, HHL_GLCM_savgh, HHL_GLCM_svarh, HHL_GLCM_senth, HHL_GLCM_dvarh, HHL_GLCM_denth, HHL_GLCM_inf1h, HHL_GLCM_inf2h, HHL_GLCM_indnc, HHL_GLCM_idmnc, HHL_GLRLM_SRE, HHL_GLRLM_LRE, HHL_GLRLM_GLN, HHL_GLRLM_RLN, HHL_GLRLM_RP, HHL_GLRLM_LGRE, HHL_GLRLM_HGRE, HHL_GLRLM_SRLGE, HHL_GLRLM_SRHGE, HHL_GLRLM_LRLGE, HHL_GLRLM_LRHGE, HHL_GLRLM_GLV, HHL_GLRLM_RLV, HHL_GLSZM_SZE, HHL_GLSZM_LZE, HHL_GLSZM_GLN, HHL_GLSZM_ZSN, HHL_GLSZM_ZP, HHL_GLSZM_LGZE, HHL_GLSZM_HGZE, HHL_GLSZM_SZLGE, HHL_GLSZM_SZHGE, HHL_GLSZM_LZLGE, HHL_GLSZM_LZHGE, HHL_GLSZM_GLV, HHL_GLSZM_ZSV, HHL_Coarseness, HHL_Contrast, HHL_Busyness, HHL_Complexity, HHL_Strength
**HLH (high, low, high)**	HLH_GLCM_autoc, HLH_GLCM_contr, HLH_GLCM_corrm, HLH_GLCM_corrp, HLH_GLCM_cprom, HLH_GLCM_cshad, HLH_GLCM_dissi, HLH_GLCM_energ), HLH_GLCM_entro, HLH_GLCM_homom, HLH_GLCM_homop, HLH_GLCM_maxpr, HLH_GLCM_sosvh, HLH_GLCM_savgh, HLH_GLCM_svarh, HLH_GLCM_senth, HLH_GLCM_dvarh, HLH_GLCM_denth, HLH_GLCM_inf1h, HLH_GLCM_inf2h, HLH_GLCM_indnc, HLH_GLCM_idmnc, HLH_GLRLM_SRE, HLH_GLRLM_LRE, HLH_GLRLM_GLN, HLH_GLRLM_RLN, HLH_GLRLM_RP, HLH_GLRLM_LGRE, HLH_GLRLM_HGRE, HLH_GLRLM_SRLGE, HLH_GLRLM_SRHGE, HLH_GLRLM_LRLGE, HLH_GLRLM_LRHGE, HLH_GLRLM_GLV, HLH_GLRLM_RLV, HLH_GLSZM_SZE, HLH_GLSZM_LZE, HLH_GLSZM_GLN, HLH_GLSZM_ZSN, HLH_GLSZM_ZP, HLH_GLSZM_LGZE, HLH_GLSZM_HGZE, HLH_GLSZM_SZLGE, HLH_GLSZM_SZHGE, HLH_GLSZM_LZLGE, HLH_GLSZM_LZHGE, HLH_GLSZM_GLV, HLH_GLSZM_ZSV, HLH_Coarseness, HLH_Contrast, HLH_Busyness, HLH_Complexity,

	HLH_Strength
**LHH (low, high, high)**	LHH_GLCM_autoc, LHH_GLCM_contr, LHH_GLCM_corrm, LHH_GLCM_corrp, LHH_GLCM_cprom, LHH_GLCM_cshad, LHH_GLCM_dissi, LHH_GLCM_energ), LHH_GLCM_entro, LHH_GLCM_homom, LHH_GLCM_homop, LHH_GLCM_maxpr, LHH_GLCM_sosvh, LHH_GLCM_savgh, LHH_GLCM_svarh, LHH_GLCM_senth, LHH_GLCM_dvarh, LHH_GLCM_denth, LHH_GLCM_inf1h, LHH_GLCM_inf2h, LHH_GLCM_indnc, LHH_GLCM_idmnc, LHH_GLRLM_SRE, LHH_GLRLM_LRE, LHH_GLRLM_GLN, LHH_GLRLM_RLN, LHH_GLRLM_RP, LHH_GLRLM_LGRE, LHH_GLRLM_HGRE, LHH_GLRLM_SRLGE, LHH_GLRLM_SRHGE, LHH_GLRLM_LRLGE, LHH_GLRLM_LRHGE, LHH_GLRLM_GLV, LHH_GLRLM_RLV, LHH_GLSZM_SZE, LHH_GLSZM_LZE, LHH_GLSZM_GLN, LHH_GLSZM_ZSN, LHH_GLSZM_ZP, LHH_GLSZM_LGZE, LHH_GLSZM_HGZE, LHH_GLSZM_SZLGE, LHH_GLSZM_SZHGE, LHH_GLSZM_LZLGE, LHH_GLSZM_LZHGE, LHH_GLSZM_GLV, LHH_GLSZM_ZSV, LHH_Coarseness, LHH_Contrast, LHH_Busyness, LHH_Complexity, LHH_Strength
**HHH (high, high, high)**	HHH_GLCM_autoc, HHH_GLCM_contr, HHH_GLCM_corrm, HHH_GLCM_corrp, HHH_GLCM_cprom, HHH_GLCM_cshad, HHH_GLCM_dissi, HHH_GLCM_energ), HHH_GLCM_entro, HHH_GLCM_homom, HHH_GLCM_homop, HHH_GLCM_maxpr, HHH_GLCM_sosvh, HHH_GLCM_savgh, HHH_GLCM_svarh, HHH_GLCM_senth, HHH_GLCM_dvarh, HHH_GLCM_denth, HHH_GLCM_inf1h, HHH_GLCM_inf2h, HHH_GLCM_indnc, HHH_GLCM_idmnc, HHH_GLRLM_SRE, HHH_GLRLM_LRE, HHH_GLRLM_GLN, HHH_GLRLM_RLN, HHH_GLRLM_RP, HHH_GLRLM_LGRE, HHH_GLRLM_HGRE, HHH_GLRLM_SRLGE, HHH_GLRLM_SRHGE, HHH_GLRLM_LRLGE, HHH_GLRLM_LRHGE, HHH_GLRLM_GLV, HHH_GLRLM_RLV, HHH_GLSZM_SZE, HHH_GLSZM_LZE, HHH_GLSZM_GLN, HHH_GLSZM_ZSN, HHH_GLSZM_ZP, HHH_GLSZM_LGZE, HHH_GLSZM_HGZE, HHH_GLSZM_SZLGE, HHH_GLSZM_SZHGE, HHH_GLSZM_LZLGE, HHH_GLSZM_LZHGE, HHH_GLSZM_GLV, HHH_GLSZM_ZSV, HHH_Coarseness, HHH_Contrast, HHH_Busyness, HHH_Complexity, HHH_Strength

V. The detailed descriptions of clinical net benefit, the "treat-all plan", and the "treat-none plan"

The net benefit was defined using the following formula:

$$Net\ benefit = \frac{TPR}{N} - \frac{FPR}{N} \times \frac{P_t}{1 - P_t}$$

In this formula, N was the sample size, P_t was the threshold probability to stratify patients as the predicted synchronous lymph node metastasis (LNM) or non-LNM. Patients with the predicted LNM probabilities greater than P_t were predicted as synchronous LNM, while patients with the predicted LNM probabilities lower than P_t were predicted as non-LNM. For patients with predicted synchronous LNM, the lymph node dissection (LND) were recommended. While for patients with predicted non-LNM, the LND was not

recommended. *TPR* was the true positive rate. *TPR* was defined as the ratio of patients with predicted synchronous LNM in the patients with actual LNM. *FPR* was the false positive rate. *FPR* was defined as the ratio of patients with predicted synchronous LNM in the patients without LNM.

The "treat-none plan" was defined that no patients were predicted as LNM. In this case, the *TPR* and *FPR* equaled to zero, and the net benefit was zero. The "treat-all plan" was defined that all patients were predicted as LNM. In this case, the *TPR* and *FPR* equaled to one, and the net benefit calculation formula was changed:

$$Net\ benefit_{treat-all\ plan} = \frac{1 - 2 \times P_t}{N \times (1 - P_t)}$$

VI. Demographic comparison of baseline clinical features between the training and validation groups

While a temporal interval existed between the training and validation groups, there were no significant differences in the baseline clinical features between the training group and the validation group neither for patients with LNM (P = 0.9773 for age, 0.0923 for gender, 0.4419 for primary hepatic lobe site, 0.9590 for number of the primary tumors 0.9464 for hepatitis, 0.9044 for cirrhosis, 0.8684 for cholelithiasis, 0.1904 for CA 19-9 level, 0.4974 for CEA level, 0.4419 for the MR-reported LNM factor) and patients with non-LNM (P = 0.8829 for age, 0.1900 for gender, 0.3374 for primary hepatic lobe site, 0.6015 for number of the primary tumors 0.8749 for hepatitis, 0.9202 for cirrhosis, 0.8050 for cholelithiasis, 0.0525 for CA 19-9 level, 0.5401 for CEA level, 0.5523 for the MR-reported LNM factor). Thus, the baseline clinical features for patients in the training and validation groups justify their use as the training and validation groups.

VII. Calculation formulas for SVM model and combination nomogram

$$SVM\ score = 0.3386 + 0.0988 \times HLH\_GLCM\_maxpr - 0.1524 \times LLH\_GLCM\_sosvh - 0.2111 \times HLL\_GLCM\_corrm - 0.4333 \times LLL\_GLCM\_denth - 0.2087 \times HLL\_GLSZM\_LGZE$$

$$Nomogram\ score = -3.4872 + 4.1198 \times SVM\ score + 1.4461 \times CA\ 19\text{-}9\ level + 1.0490 \times MR\text{-}reported\ LNM$$

VIII. Predictive performances of different feature selection methods

To find the optimal feature selection algorithm for the problem here, we compared the performances of several feature selection methods, including mRMR, least absolute shrinkage and selection operator (LASSO), Random forest, Elastic net, Wilcoxon, and Gini index, and the results were summarized in Table S5 below. In the training group, the P values calculated based on the Delong test showed that the mRMR, LASSO, Elastic net, and Random forest were all less than 0.0001. Using the AUC value as an evaluation index, the mRMR method and LASSO method showed the best performances. In the validation group, the P value for the mRMR method was the lowest, while its AUC was the highest. Therefore, the mRMR was chosen as the optimal method in this paper.

Table S5. Predictive performances of different feature selection methods

Methods	Training group				Validation group			
	Sensitivity	Specificity	AUC (95% CI)	*P*	Sensitivity	Specificity	AUC (95% CI)	*P*
mRMR	65.96%	79.66%	0.788 (0.698-0.862)	<0.0001	52.63%	91.30%	0.787 (0.634-0.898)	<0.0001
LASSO	70.21%	71.19%	0.773 (0.692-0.857)	<0.0001	63.16%	60.87%	0.714 (0.554-0.843)	0.0077
Elastic Net	82.98%	55.93%	0.737 (0.643-0.818)	<0.0001	73.68%	43.48%	0.629 (0.467-0.773)	0.1385
Random Forest	65.96%	79.66%	0.759 (0.666-0.837)	<0.0001	42.11%	73.91%	0.673 (0.511-0.809)	0.0396
Wilcoxon	57.45%	79.66%	0.689 (0.592-0.775)	0.0004	57.89%	78.26%	0.693 (0.532-0.826)	0.0238
Gini Index	49.15%	80.85%	0.679 (0.581-0.766)	0.0006	43.48%	78.95%	0.719 (0.559-0.846)	0.0074

Note: AUC: area under the curve; CI: confidence interval. *P* value was calculated using the Delong test.

IX. Histograms regarding the distributions of AUCs for the SVM mode and combination nomogram

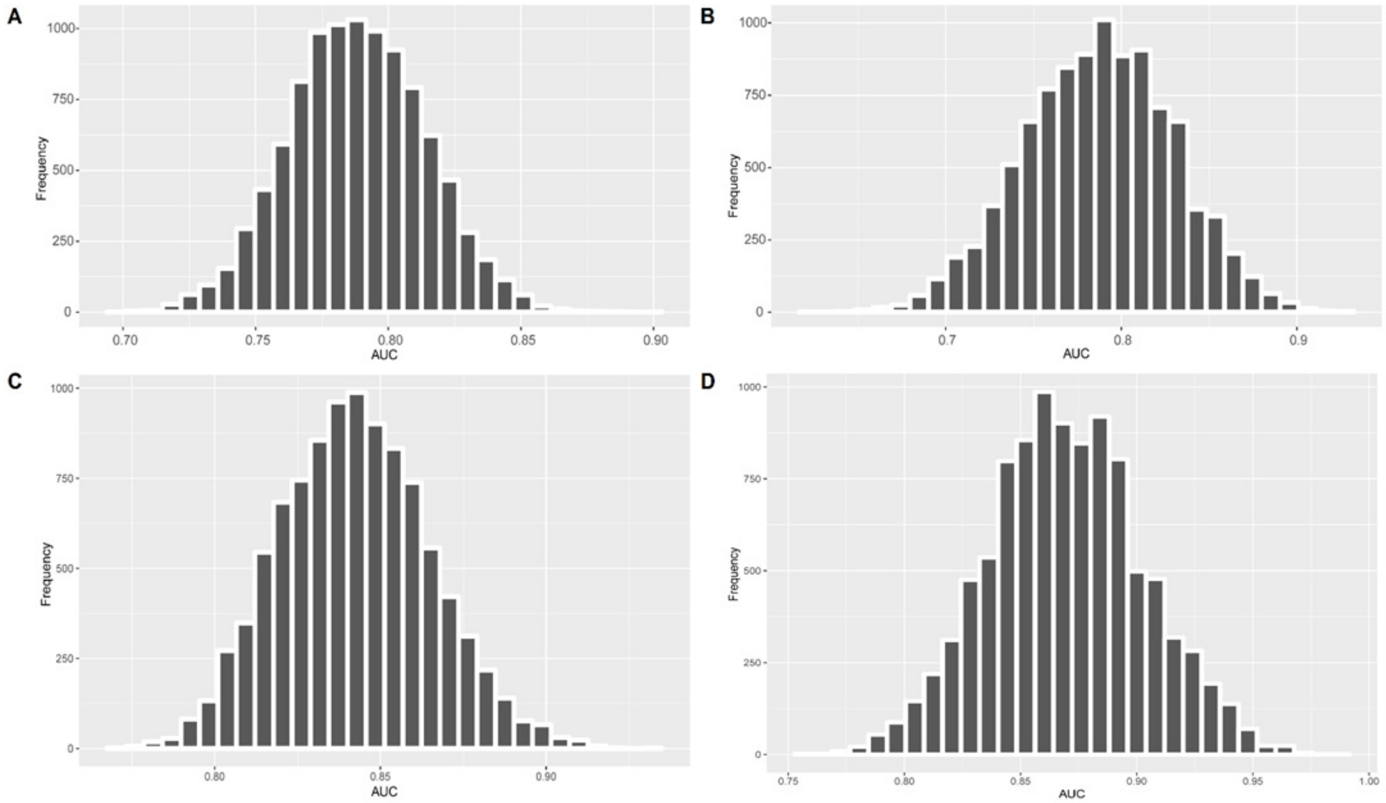

Figure S2. Histograms regarding the distributions of AUCs from the bootstrap method for SVM model and combination nomogram in both training and validation groups. (A) Histogram for SVM model in the training group; (B) histogram for SVM model in the validation group; (C) histogram for combination nomogram in the training group; (B) histogram for combination nomogram in the validation group.

X. The multivariable analysis for model construction

In the multivariable analysis, we used the Akaike information criterion (AIC) and the independence analysis to select the optimal factors. The detailed AIC values in the model construction procedure were showed in Table S6. Firstly, a combination with the minimum AIC value of 117.49 was selected, involving SVM score, CA 19-9 level, number of the primary tumors, primary hepatic lobe site, and the MR-reported LNM factor was selected. By using the independence analysis for features in the model, three features of SVM score, CA 19-9 level, and the MR-reported LNM factor were reported independent with P-values < 0.05, while two features of primary hepatic lobe site and number of the primary tumors were reported non-independent with P-values > 0.05. Table S8 showed the P-values for these five features. Then, we removed these two redundant features and construct a new model with the independent features only. Table S9 showed the P-values for the selected factors in the new prediction model. Thus, we used the model with the SVM score,

CA 19-9 level, and the MR-reported LNM factor in this study.

Table S6. AIC value changes in the model construction

Variable	*AIC*
SVM score & Gender & Age & Cholelithiasis & Hepatitis B & Cirrhosis & Primary hepatic lobe site & Number of the primary tumors &CA 19-9 level & CEA level & MR-reported LNM	127.41
SVM score & Gender & Age & Cholelithiasis & Cirrhosis & Primary hepatic lobe site & Number of the primary tumors &CA 19-9 level & CEA level & MR-reported LNM	125.43
SVM score & Gender & Age & Cholelithiasis & Cirrhosis & Primary hepatic lobe site & Number of the primary tumors &CA 19-9 level & MR-reported LNM	123.45
SVM score & Gender & Age & Cholelithiasis & Primary hepatic lobe site & Number of the primary tumors &CA 19-9 level & MR-reported LNM	121.53
SVM score & Age & Cholelithiasis & Primary hepatic lobe site & Number of the primary tumors &CA 19-9 level & MR-reported LNM	119.76
SVM score & Cholelithiasis & Primary hepatic lobe site & Number of the primary tumors &CA 19-9 level & MR-reported LNM	118.19
SVM score & Primary hepatic lobe site & Number of the primary tumors &CA 19-9 level & MR-reported LNM	117.49

Note: SVM, support vector machine; LNM, lymph node metastasis; CA19-9, serum carbohydrate antigen 19-9; CEA, serum carcinoembryonic antigen.

Table S7. VIFs for all the candidate variables in the logistic regression analysis

Variable	VIF
SVM score	5.9610
Gender	2.3877
Age	47.8974
Cholelithiasis	1.4593
Hepatitis B	1.6686
Cirrhosis	1.0680
Primary hepatic lobe site	1.8118
Number of the primary tumors	1.4319
CA 19-9 level	4.1702
CEA level	1.9743
MR-reported LNM	0.0772

Note: LNM, lymph node metastasis; CA19-9, serum carbohydrate antigen 19-9; CEA, serum carcinoembryonic antigen.

Table S8. Multivariable analysis for five features selected

Variable	*P*
SVM score	0.0003
CA 19-9 level	0.0078
MR-reported LNM	0.0249
Primary hepatic lobe site	0.0546
Number of the primary tumors	0.0772

Note: LNM: lymph node metastasis; CA19-9: serum carbohydrate antigen 19-9.

Table S9. Multivariable analysis for features used in the nomogram

Variable	Coefficients	*P*	OR (95% CI)
SVM score	4.1198	<0.0001	61.5448 (7.8097-485.0073)
CA 19-9 level	1.4461	0.0081	4.2467 (1.4569-12.3785)
MR-reported LNM	1.0490	0.0307	2.8548 (1.1022-7.3941)

Note: SVM, support vector machine; OR, odds ratio; CI, confidence interval.

Reference:

1. Haralick RM, Shanmugam K. Textural features for image classification. IEEE Trans Syst Man Cybern. 1973; SMC-3: 610-21.
2. Galloway MM. Texture analysis using gray level run lengths. Comput Graph Image Process. 1975; 4: 172-9.
3. Amadasun M, King R. Textural features corresponding to textural properties. IEEE Trans Syst Man Cybern. 1989; 19: 1264-74.
4. Chu A, Sehgal CM, Greenleaf JF. Use of gray value distribution of run lengths for texture analysis. Pattern Recognit Lett. 1990; 11: 415-9.
5. Dasarathy BV, Holder EB. Image characterizations based on joint gray level-run length distributions. Pattern Recognit Lett. 1991; 12: 497-502.
6. Thibault G, Fertil B, Navarro C, L., Pereira S, Cau P, Lévy N, et al. Texture indexes and gray level size zone matrix: application to cell nuclei classification. Pattern Recognition Inf Process. 2009; 140-5.

7. Vallieres M, Freeman CR, Skamene SR, El Naqa I. A radiomics model from joint FDG-PET and MRI texture features for the prediction of lung metastases in soft-tissue sarcomas of the extremities. Phys Med Biol. 2015; 60: 5471-96.

8. Vickers AJ, Elkin EB. Decision curve analysis: a novel method for evaluating prediction models. Med Decis Making. 2006; 26: 565-74.

9. Vickers AJ, Van Calster B, Steyerberg EW. Net benefit approaches to the evaluation of prediction models, molecular markers, and diagnostic tests. BMJ. 2016; 352: 5.

10. Coroller TP, Grossmann P, Hou Y, Velazquez ER, Leijenaar RTH, Hermann G, et al. CT-based radiomic signature predicts distant metastasis in lung adenocarcinoma. Radiother Oncol. 2015; 114: 345-50.

11. Parmar C, Grossmann P, Bussink J, Lambin P, Aerts H. Machine learning methods for quantitative radiomic biomarkers. Sci Rep. 2015; 5: 11.

12. Huang Y, Liang C, He L, Tian J, Liang C, Chen X, et al. Development and validation of a radiomics nomogram for preoperative prediction of lymph node metastasis in colorectal cancer. J Clin Oncol. 2016; 34: 2157-64.

13. Zhang B, He X, Ouyang F, Gu D, Dong Y, Zhang L, et al. Radiomic machine-learning classifiers for prognostic biomarkers of advanced nasopharyngeal carcinoma. Cancer Lett. 2017; 403: 21-7.

14. Li H, Galperin-Aizenberg M, Pryma D, Simone CB, Fan Y. Unsupervised machine learning of radiomic features for predicting treatment response and overall survival of early stage non-small cell lung cancer patients treated with stereotactic body radiation therapy. Radiother Oncol. 2018; 129: 218-26.

15. Akaike H. A new look at the statistical model identification. IEEE Trans Automat Contr. 1974; 19: 716-23.

16. Wu S, Zheng J, Li Y, Yu H, Shi S, Xie W, et al. A radiomics nomogram for the preoperative prediction of lymph node metastasis in bladder cancer. Clin Cancer Res. 2017; 23: 6904-11.
